# Experimental and Simulation Studies of Imidazolium Chloride Ionic Liquids with Different Alkyl Chain Lengths for Viscosity Reductions in Heavy Crude Oil: The Effect on Asphaltene Dispersion

**DOI:** 10.3390/molecules29051184

**Published:** 2024-03-06

**Authors:** Chaoyue Xiang, Yangwen Zhu, Guanghao Liu, Tao Liu, Xinru Xu, Jingyi Yang

**Affiliations:** 1 International Joint Research Center of Green Energy Chemical Engineering, East China University of Science and Technology, Meilong Road 130, Shanghai 200237, China; 2 Petroleum Exploration and Production Research Institute, SINOPEC, Beijing 100083, China; 3 Shanghai Key Laboratory of Multiphase Materials Chemical Engineering, School of Chemical Engineering, East China University of Science and Technology, Shanghai 200237, China

**Keywords:** ionic liquids, heavy crude oil, asphaltenes, alkyl chain lengths, molecular simulation

## Abstract

Heavy crude oil poses challenges in terms of extraction and transportation due to its high viscosity. In the pursuit of effective methods to reduce viscosity in heavy crude oil, this study investigates the potential of imidazolium chloride ionic liquids with varying alkyl chain lengths as viscosity reducers. The experimental results demonstrate that the addition of 1-dodecyl-3-methylimidazole chloride ([C12-MIM]Cl) leads to a maximum viscosity reduction of 49.87%. Solubility parameters were calculated based on characterization of the average molecular structure of the asphaltenes. The viscosity reduction effect is enhanced when the solubility parameter of the ionic liquid closely matches that of the asphaltene. The initial asphaltene deposition point of heavy crude oil is increased from 63% to 68% with the addition of 150 mg/L [C12-MIM]Cl. Furthermore, the average particle size of asphaltene deposits decreases from 79.35 μm to 48.54 μm. The viscosity of heavy crude oil is influenced by the aggregation of asphaltenes. The ability of ionic liquids, especially those with longer alkyl chains, to disperse asphaltene molecules and reduce viscosity has been confirmed through molecular dynamics and quantum mechanical simulations.

## 1. Introduction

Petroleum is a vital resource in modern society, playing an indispensable role in a wide range of activities, including transportation and industrialization. As the extraction of light crude oil continues, the importance of heavy crude oil resources has increased, but its high viscosity presents challenges for extraction and transportation [1]. A variety of methods are employed to mitigate the viscosity of heavy crude oil, including emulsification, heating, and dilution [2,3,4]. However, these approaches have inherent drawbacks, such as high costs, excessive energy consumption, and the need for subsequent processing. Asphaltenes, the most polar components of crude oil, are considered to be the main cause of the high viscosity of heavy crude oil [5]. Chemical viscosity reducers that operate through the interaction and dispersion of asphaltene molecules have garnered significant attention. Dispersant molecules serve to stabilize and disperse asphaltenes in crude oil, impeding the formation of aggregates [6,7,8,9,10]. The dispersion of asphaltene aggregates results in a significant reduction in viscosity by decreasing the volume of the dispersed phase and increasing the volume of the continuous phase in crude oil [11].

Ionic liquids, which exhibit amphiphilicity, have promising applications as functionalized molecules for reducing viscosity in heavy oils. The advantages of ionic liquids are their environmental friendliness, low vapor pressure, and remarkable thermal and chemical stability, characterized by a melting point below 100 °C [12,13,14]. Changing the anion and cation components of ionic liquids changes their chemical and thermomechanical properties, enabling them to be used in a wide range of applications [15,16]. In recent years, ionic liquids have attracted considerable interest due to their unique physicochemical properties, and have found diverse applications in the petroleum industry, including asphaltene dispersion, enhanced oil recovery, demulsification processes, and solvent extraction [17,18,19,20,21].

Extensive research has been conducted on the application of ionic liquids as viscosity reducers for heavy oil. Atta et al. [22] prepared three different imidazolium-based ionic liquids with various organic salt anions. Among them, imidazolium ionic liquid with an alicyclic hydroxybenzene ring had the highest degree of hydrophobicity and demonstrated superior performance in dispersing asphaltenes and achieving significant viscosity reduction. Baghersaei et al. [23] found that ionic liquids primarily stabilize asphaltenes through π–π and electrostatic interactions. The efficiency of asphaltene dispersion is directly related to the net negative anionic charge of the ionic liquid. Subramanian et al. [24] investigated the impact of various ionic liquids on reducing the viscosity of heavy crude oil and bitumen. They observed that even at low concentrations, the addition of dodecylpyridinium chloride ionic liquid can reduce the viscosity of heavy oil by 35%. Shaban et al. [25] utilized ILs, specifically [BMIM][FeCl_4_], for heavy crude oil upgrading and achieved a remarkable 78.6% decrease in crude oil viscosity at 90 °C. Molecular dynamics (MD) simulations have become a widely adopted tool for studying the aggregation behavior of asphaltenes in the presence of ionic liquids [26,27,28]. El-hoshoudy et al. [29] employed MD simulations to investigate the interaction energies and intermolecular forces between different ionic liquids and asphaltene molecules. The simulations demonstrated that increasing the alkyl chain length in the dispersant molecule enhanced asphaltene dispersion. In the context of viscosity reduction in heavy crude oil, Xu et al. [30] found, through molecular dynamics simulations, that the characteristic side chain atoms in the viscosity reducer interact strongly with the asphaltene molecules, resulting in improved viscosity reduction. Additionally, Song et al. [31] conducted simulations to investigate the formation of a network between asphaltenes and resin molecules, which is considered a key factor contributing to the high viscosity of heavy crude oil.

In this study, we focused on the effectiveness of imidazolium chloride ionic liquids with different alkyl chain lengths as viscosity reducers in heavy crude oil, a topic that has received limited attention in previous research on ionic liquids dispersing asphaltenes. The aim of the study was to elucidate the mechanism of viscosity reduction by assessing the dispersion of asphaltenes. The solubility parameters were calculated to assess the compatibility between the ionic liquid and asphaltene molecules. Furthermore, we employed UV–visible spectrophotometry to determine the initial deposition point of asphaltenes in heavy crude oil. The particle size of asphaltene deposits was analyzed using a laser particle size analyzer, providing insights into the dispersion of asphaltenes facilitated by imidazolium chloride ionic liquids. Quantum mechanical (QM) simulations were used to elucidate the role of different components in [C12-MIM]Cl. The aggregation distribution of asphaltene molecules in the toluene–heptane model oil system after the addition of different ionic liquids was observed through molecular dynamics simulations. The intermolecular distances between asphaltene molecules in the simulated system were represented by radial distribution functions.

## 2. Results and Discussion

### 2.1. Heavy Oil Viscosity Reduction by Ionic Liquids

**Figure 1:**
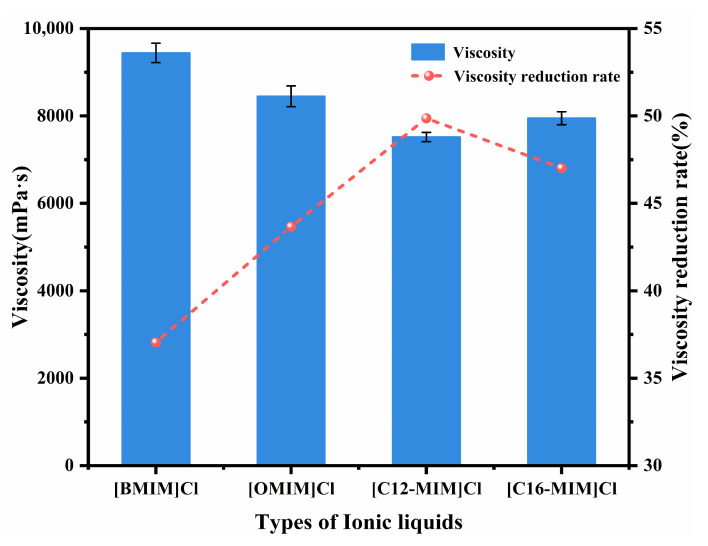
The effect of different alkyl chain lengths of imidazolium chloride ionic liquids on the viscosity of heavy crude oil.

**Figure 2:**
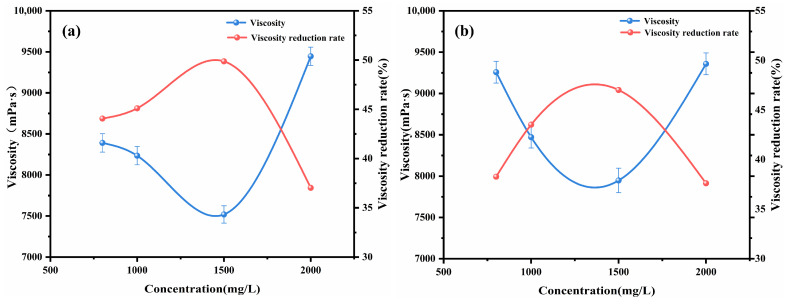
The effect of imidazolium chloride ionic liquid concentration on the viscosity of heavy crude oil: (**a**) [C12-MIM]Cl; (**b**) [C16-MIM]Cl.

The impact of alkyl tail length on crude oil viscosity was investigated by examining imidazolium chloride ionic liquids with tail lengths of C4, C8, C12, and C16. Figure 1 presents the viscosity and viscosity reduction results for Shengli heavy oil containing 1500 mg/L of the various ionic liquids at 50 °C. The maximum viscosity reduction of the crude oil was observed to be up to 49.87% with the addition of [C12-MIM]Cl. The relationship between the tail length of the ionic liquid and the viscosity reduction exhibited a non-monotonic trend. Ionic liquids with longer alkyl tail lengths demonstrated greater effectiveness in reducing the viscosity of heavy crude oil compared with those with shorter tail lengths. The research indicates that the alkyl chains of functional chemicals can create a steric hindrance to the π–π stacking of asphaltene molecules, preventing asphaltene aggregation [24,32]. From the trend observed in Figure 1, it can be seen that the longer alkyl tails of imidazolium chlorides in the ionic liquids impose stronger steric hindrance on the π–π stacking of asphaltene molecules. This leads to a reduction in asphaltene aggregation, and consequently, a greater decrease in viscosity. Furthermore, the effects of the concentrations of [C12-MIM]Cl and [C16-MIM]Cl, which exhibited excellent viscosity-reducing effects, on the viscosity reduction of heavy crude oil were investigated (as shown in Figure 2). It was observed that with an increasing concentration, the viscosity reduction of the ionic liquids initially increased and then decreased. At a concentration of 1500 mg/L, [C12-MIM]Cl and [C16-MIM]Cl displayed the most significant viscosity-reducing effects. However, when the concentration of the ionic liquid exceeded 1500 mg/L, the rate of viscosity reduction in heavy crude oil decreased. This decrease in effectiveness can be attributed to the aggregation of the ionic liquids themselves, which weakens their dispersing effect on asphaltene, and consequently reduces the overall viscosity reduction efficiency.

### 2.2. Characterization of Asphaltene Structure

To investigate the mechanism of viscosity reduction by ionic liquids, the asphaltene fraction of the heavy oil used was characterized using elemental analysis, Fourier transform infrared spectroscopy, ^1^H nuclear magnetic resonance, and ^13^C nuclear magnetic resonance. The experimental results are presented in Text S2 (Supplementary Materials). The average molecular structural parameters of the asphaltene fraction were calculated by utilizing the ^1^H-NMR/IR method based on the obtained characterization results [33]. The calculated results are presented in Table 1.

**Figure 3:**
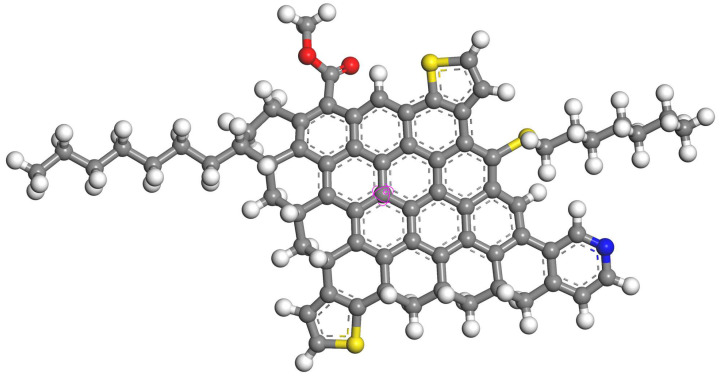
Average structure of asphaltene (carbon atoms in gray; hydrogen atoms in white; oxygen atoms in red; sulfur atoms in yellow; nitrogen atoms in blue; the aromatic carbon atoms marked in purple are set as central atoms).

Table 2 shows the forms of oxygen, sulfur, and nitrogen present in the asphaltene using X-ray photoelectron spectroscopy. In asphaltene, oxygen primarily exists in the form of single-bonded oxygen and carboxyl groups, with a small number of carbonyl groups. Sulfur in asphaltenes mainly exists in the forms of thioethers and thiophenes, while some sulfur exists in the form of sulfoxides. Nitrogen primarily exists in asphaltene in the form of pyridine and pyrrole. The nitrogen content of pyridine is slightly higher than that of pyrrole. By combining the results of XPS analysis with the calculated average molecular structure parameters, the average molecular structure of asphaltene was determined. The established average molecular structure of asphaltene is depicted in Figure 3.

### 2.3. The Effect of Solubility Parameters on the Viscosity Reduction

The solubility parameter (δ) is used to correlate the phase stability of crude oil. It has been extensively used to model the aggregation and deposition of asphaltene during production and transportation processes [34]. Based on the molecular model of asphaltene, the solubility parameter of asphaltene was calculated to be 20.60 MPa^1/2^ using Wang’s method [35].

The solubility parameters of the ionic liquids used for viscosity reduction are presented in Table 3. The solubility parameters of [BMIM]Cl were obtained from the literature [36], while the solubility parameters of the other ionic liquids were calculated using the group contribution method [37]. The detailed methods are presented in Text S3 (Supplementary Materials). The solubility parameters of the ionic liquids approached the values of asphaltenes in the following order: [C12-MIM]Cl > [C16-MIM]Cl > [OMIM]Cl > [BMIM]Cl. This trend is consistent with their viscosity reduction effect. When two substances have similar solubility parameters, they exhibit a strong propensity to interact and dissolve in each other. The solubility parameter of [C12-MIM]Cl was found to be the closest to that of asphaltene, suggesting that it exhibited the strongest interaction and solubility with asphaltene, thereby possessing the greatest stabilization capability.

### 2.4. Dispersion of Asphaltenes by Ionic Liquids

**Figure 4:**
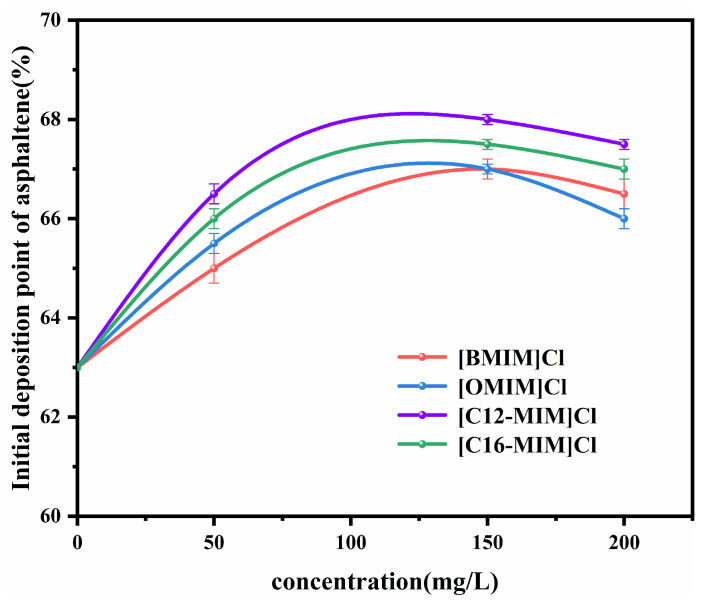
Effect of ionic liquid concentration on the deposition point of asphaltene with different alkyl chain lengths.

According to the Supplementary Materials (S1), the initial deposition point of asphaltenes in heavy crude oil solution (1800 mg/L) was 63 vol% without the addition of ionic liquids. Figure 4 illustrates the initial deposition points of asphaltenes in heavy crude oil solutions after the addition of imidazolium chloride ionic liquids with different alkyl chain lengths. The inclusion of ionic liquids resulted in an increase in the amount of n-heptane required to induce asphaltene precipitation. This indicates that the ionic liquids were able to improve the stability of the system. The trend observed for the asphaltene deposition point with respect to the ionic liquid concentration is consistent with that of the viscosity reduction rate with an increasing ionic liquid concentration. With the addition of 150 mg/L [C12-MIM]Cl, the initial asphaltene deposition point increased from 63 vol% to 68 vol%. It is important to note that ionic liquids have limited solubility in heavy crude oil solutions, and an excessive amount of ionic liquid can lead to self-aggregation. This aggregation reduces the amount of ionic liquid available to stabilize asphaltene. Therefore, at a concentration of 200 mg/L, the initial deposition point of asphaltene is slightly reduced.

**Figure 5:**
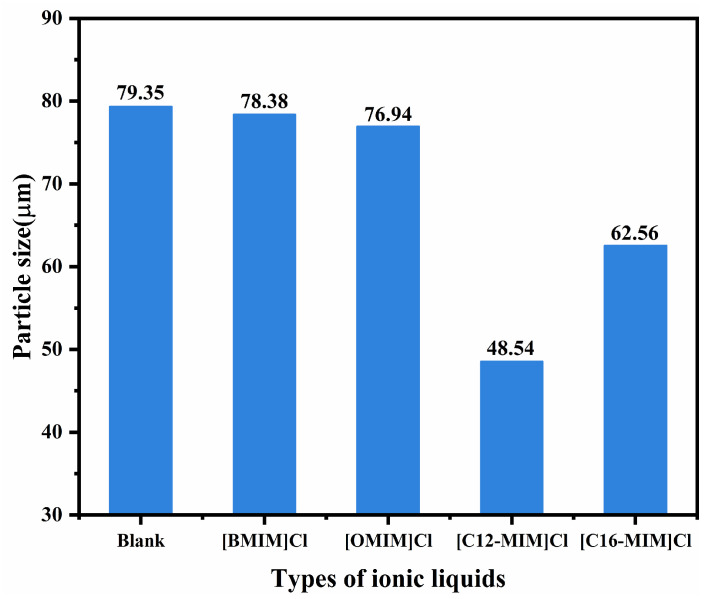
Average particle size of asphaltene sediment particles.

To investigate the dispersion of asphaltenes by ionic liquids, we examined the sediment particle size of asphaltenes near the initial deposition point. The experimental results are presented in Text S4 (Supplementary Materials). Figure 5 presents the average particle size of asphaltene deposits after the addition of ionic liquids with varying alkyl chain lengths. The inclusion of [BMIM]Cl and [OMIM]Cl led to a slight decrease in the average particle size of asphaltene deposits. On the other hand, the addition of [C12-MIM]Cl and [C16-MIM]Cl resulted in a significant reduction in the average size of asphaltene sediment particles. Notably, the largest reduction in average asphaltene sediment particle size, from 79.35 μm to 48.54 μm, was observed with the addition of [C12-MIM]Cl. These findings indicate that imidazolium chloride ionic liquids possess the ability to inhibit asphaltene deposition and reduce the size of asphaltene sediment particles, thereby exhibiting a favorable dispersing effect on asphaltenes. Moreover, imidazolium chloride ionic liquids with longer alkyl chains demonstrate superior asphaltene dispersion compared with those with shorter alkyl chains. This increased effectiveness in dispersing asphaltenes can be attributed to the spatial resistance provided by the alkyl side chains, which enables the ionic liquids to stabilize asphaltenes. However, excessive carbon chain length can lead to crystallization of ionic liquids with waxes in crude oil, resulting in the low solubility of ionic liquids in crude oil [22]. Alkyl chains of appropriate length play a crucial role in assisting ionic liquids in the dispersion of asphaltenes. The reduction in particle size of asphaltene aggregates reduces the volume of dispersed phases in heavy oil colloids, resulting in a significant reduction in viscosity.

### 2.5. Interaction of Ionic Liquids with Asphaltenes

**Figure 6:**
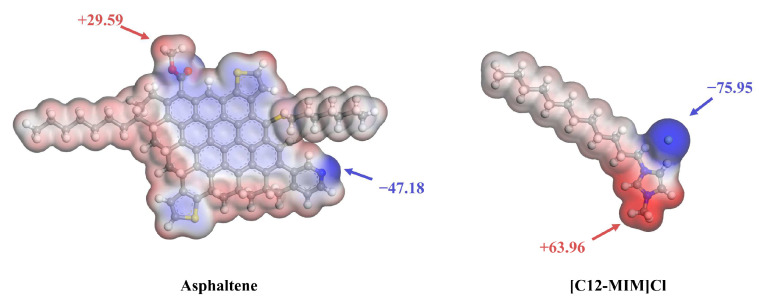
Molecular ESP maps for asphaltene and [C12-MIM]Cl.

The electrostatic potential (ESP) is an important method for characterizing non-covalent interactions, as it allows us to understand which part of a molecule serves as the target for nucleophilic or electrophilic attacks. The electrostatic potential results for asphaltene and [C12-MIM]Cl molecules are shown in Figure 6. A negative ESP is represented by the blue regions, indicating higher electron density and electron-rich areas. Conversely, a positive ESP is represented by the red regions, indicating a lower electron density and electron-deficient areas. The asphaltene has the maximum ESP value at the carboxyl hydrogen (29.59 kcal/mol) and the minimum ESP value at the pyridine nitrogen (−47.18 kcal/mol). The [C12-MIM]Cl molecule has the maximum ESP at the hydrogen on the imidazolium cyclomethyl group (63.96 kcal/mol) and the minimum ESP at the chloride ion (−75.95 kcal/mol). The [C12-MIM]Cl alkyl chain exhibits an ESP value indicating a lower electron density, which suggests a decreased tendency to interact with other groups. The results indicate that the cationic groups and anions of [C12-MIM]Cl readily adsorb with the polar heteroatoms of the asphaltenes. The alkyl chain of [C12-MIM]Cl has a weak interaction with other components and inhibits asphalt aggregation by creating spatial resistance.

### 2.6. Spatial Distribution of Asphaltene Molecules

**Figure 7:**
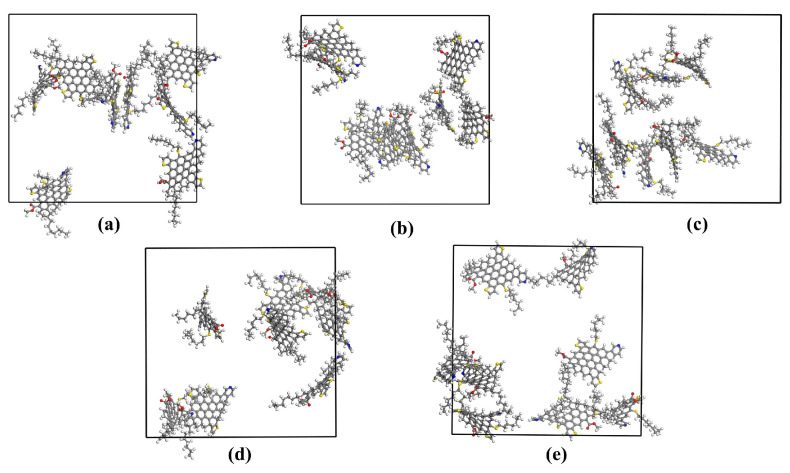
Molecular dynamics simulation of the last frame conformation: (**a**) the blank group; (**b**) with the addition of [BMIM]Cl; (**c**) with the addition of [OMIM]Cl; (**d**) with the addition of [C12-MIM]Cl; and (**e**) with the addition of [C16-MIM]Cl.

The final structures of the simulation box for different ionic liquids are displayed in Figure 7. Toluene, n-heptane, and ionic liquids molecules are not shown to understand the asphaltene aggregation phenomena better. In investigations of asphaltene aggregates, three methods are commonly employed to calculate intermolecular distances: (a) the distance between the closest atoms of two molecules; (b) the distance between specific atoms in two molecules; and (c) the distance between the centers of mass of two molecules. After selecting the calculation method, a specific cut-off interval is set. In this study, we utilized the distance between the closest atoms method to determine asphaltene aggregation, with a cut-off threshold of 0.35 nm chosen for the investigation of asphaltene aggregation [28]. As depicted in Section 4, the initial structures of each simulated system consisted of eight asphaltene monomers. At the end of the simulation, in the system without ionic liquids, asphaltene existed as a monomer and a heptamer. Upon the addition of [BMIM]Cl, the arrangement consisted of a dimer and a hexamer. Similarly, the addition of [OMIM]Cl resulted in an arrangement comprising a trimer and a pentamer. The most effective dispersion was achieved with the addition of [C12-MIM]Cl, where the arrangement consisted of two monomers, a dimer, and a tetramer. Finally, when [C16-MIM]Cl was added, the arrangement consisted of one dimer and two trimers. The results clearly demonstrate that chlorinated imidazolium ionic liquids induce alterations in asphaltene aggregation (Figure 7).

### 2.7. Radial Distribution Function

**Figure 8:**
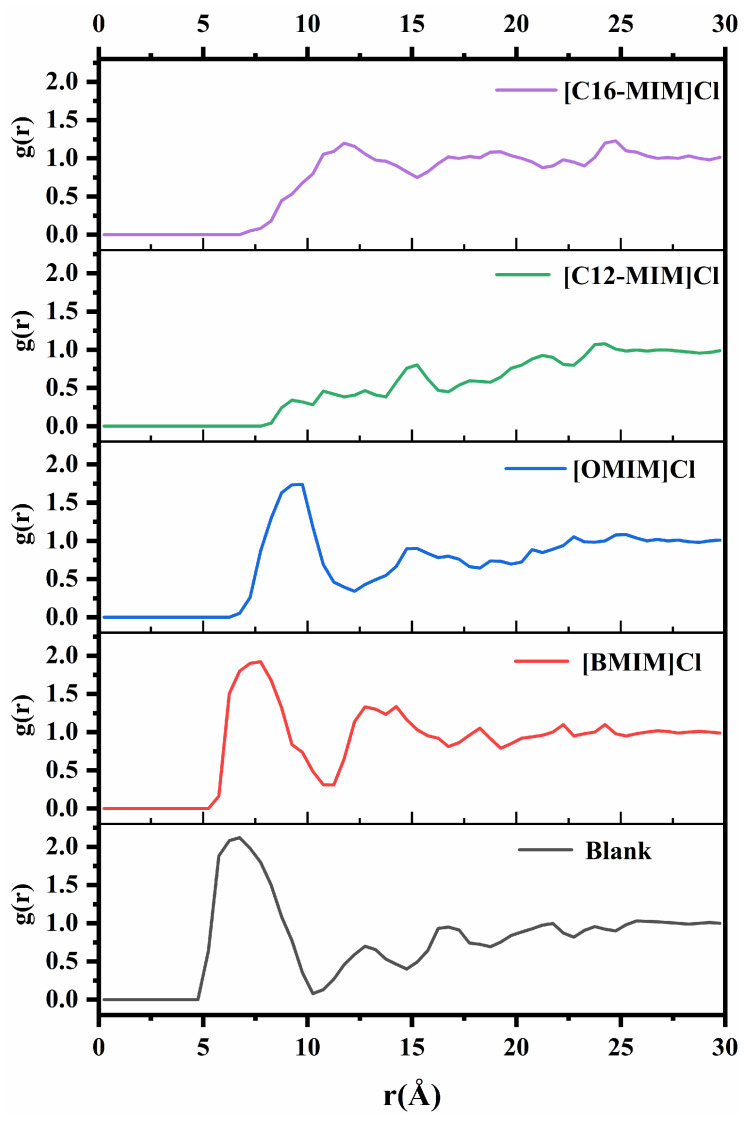
Radial distribution functions between asphaltene center atoms in different systems.

To further investigate the interaction between ionic liquid molecules and asphaltene molecules, the radial distribution function (RDF) between the central atoms of the asphaltene molecule was examined. The carbon atom captured on the aromatic ring, as shown in Figure 3, was selected as the central atom. Figure 8 presents the RDF of the central atoms for different simulation systems. The main peak of the radial distribution function is located at 6.6 Å without the addition of ionic liquid. With the addition of [BMIM]Cl and [OMIM]Cl, the peaks were located at 7.5 Å and 9.4 Å, respectively. Simultaneously, the addition of ionic liquids with shorter alkyl chains resulted in a reduction in the intensity of the main peak in the radial distribution function. The radial distribution function shows a tiny peak at 15 Å when [C12-MIM]Cl is added. With the addition of [C16-MIM]Cl the peak was located at 11.9 Å. These observations indicate that (1) asphaltene molecules are further apart in the presence of ionic liquids compared with when no ionic liquids are added; and (2) imidazolium chloride ionic liquids with longer alkyl chains have a greater impact on asphaltenes compared with those with shorter alkyl chains.

### 2.8. Viscosity Reduction Mechanism

**Figure 9:**
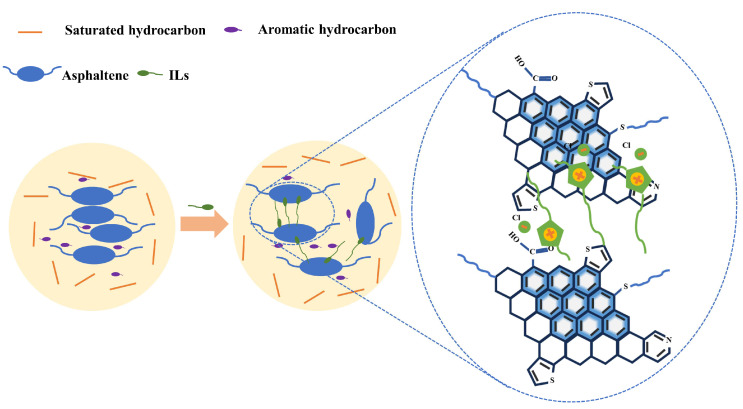
Mechanism of asphaltene dispersion by imidazolyl chloride salt ionic liquid.

Asphaltene molecules present in heavy crude oil exhibit a strong tendency to aggregate when suspended in crude oil. These asphaltene aggregates contribute to high internal friction within the crude oil system during relative displacement, consequently resulting in an elevated crude oil viscosity. Imidazolium chloride ionic liquids are adsorbed on asphaltene surfaces through electrostatic and π–π interactions, with the polyaromatic core of asphaltene and polar heteroatoms. Alkyl chains of an appropriate length can form protective layers around asphaltene molecules, effectively reducing the size of asphaltene aggregates and preventing their precipitation. The spatial mesh formed by asphaltene molecules in heavy crude oil is disrupted, resulting in a reduction in the viscosity of the heavy crude. Figure 9 presents a mechanism of asphaltene dispersion by imidazolium chloride ionic liquid.

## 3. Materials and Methods

### 3.1. Materials

The experimental study utilized Shengli heavy crude oil; its main properties are summarized in Table 4. The chemicals employed in the experiment were sourced as follows. Toluene, n-heptane, and ethanol, all of which were of analytical purity, were obtained from Shanghai Taitan Science and Technology Co. Ltd. The ionic liquids used, namely 1-butyl-3-methylimidazolium chloride ([BMIM]Cl), 1-octyl-3-methylimidazolium chloride ([OMIM]Cl), 1-dodecyl-3-methylimidazolium chloride ([C12-MIM]Cl), and 1-hexadecyl-3-methylimidazole chloride ([C16-MIM]Cl), were procured from Lanzhou Institute of Chemical Physics, Chinese Academy of Sciences. The purity of all the ionic liquids exceeded 99%.

### 3.2. Determination of the Viscosity of Heavy Crude Oil

The viscosity of the heavy crude oil was determined at a temperature of 50 °C using an NDJ-8ST viscometer, following the guidelines outlined in the ASTM D2196-2015 standard [38]. To quantify the viscosity reduction achieved, the viscosity reduction rate was calculated using Equation (1).

(1) $$f=\frac{\mu_{0}-\mu }{\mu_{0}}\times 100\%$$

where *f* is the viscosity reduction rate (%), *μ*_0_ is the heavy crude oil viscosity (mPa·s), and *μ* is the heavy crude oil viscosity after adding ionic liquids (mPa·s).

### 3.3. Test of Asphaltene Precipitation

The initial deposition point of asphaltene is used to represent the stability of asphaltene in heavy oil [39]. The initial deposition point of asphaltenes was determined by UV spectrophotometry using a 752N UV–visible spectrophotometer. The detailed methods are presented in Text S1 (Supplementary Materials).

### 3.4. Particle Size Determination

To a 10 ml volumetric flask, 9 mL of a 2000 mg/L heavy crude oil toluene-n-heptane solution (toluene/n-heptane, 3:7 by volume) and 0.5 mL of a 3000 mg/L ethanol solution in ionic liquid were added, and the volume was then fixed with ethanol. A solution with a heavy crude oil concentration of 1800 mg/L and an ionic liquid concentration of 150 mg/L was obtained. The solution was left at 25 °C for 14 h, and the particle size and distribution of the asphaltene sediment particles were measured using the LS 13 320 laser particle size analyzer produced by the American Beckman Coulter company.

### 3.5. Characterization of Asphaltenes

Asphaltenes were separated according to ASTM D6560-2000 [40]. The elemental composition of the asphaltene, including carbon, hydrogen, sulfur, and nitrogen, was determined using a Vario Micro Cube Elemental Analyzer (Elementar Company, Langenselbold, Germany). The oxygen content was obtained using the subtractive difference method. X-ray photoelectron spectroscopy of the asphaltene was performed using an ESCALAB 250Xi spectrometer (Thermo Fischer Scientific, Waltham, MA, USA). The mean molecular weight and distribution of the asphaltenes were determined using tetrahydrofuran (THF) as the mobile phase on a Waters 1515 gel permeation chromatograph (WATERS, Milford, MA, USA). The ^1^H NMR and ^13^C NMR spectra of the asphaltenes were obtained using an Ascend 400MHz nuclear magnetic resonance (NMR) spectrometer (BRUKER, Berlin, Germany). Deuterated chloroform (CDCl3) was used as the solvent, and tetramethylsilane (TMS) served as the internal standard. Fourier-transform infrared spectroscopy (FT-IR) analysis of the asphaltene was performed using a Nicolet 6700 Fourier transform infrared spectrometer (Thermo Fisher Scientific, Waltham, MA, USA).

## 4. Molecular Dynamics Simulation Settings

To investigate the viscosity reduction mechanism of ionic liquids at the molecular scale, quantum mechanical simulations were performed using the DMOL3 program in Materials Studio 6.1 software. The calculations used double numerical basis sets plus polarization functional (DNP), which offer both high computational accuracy and the ability to simulate large molecules such as asphaltenes [41]. The Becke–Lee–Yang–Parr (BLYP) function with generalized gradient approximation (GGA) was chosen to calculate the electrostatic potentials of molecules.

**Figure 10:**
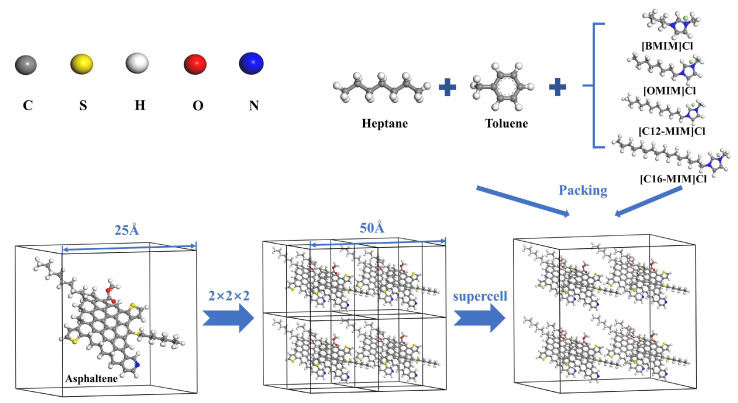
Graphical illustration of molecular dynamics modeling.

The MD simulation process was conducted using Materials Studio 6.1 software, employing the COMPASS forcefield. N-heptane and toluene molecules were chosen as solvents to replace the light crude oil. The asphaltene model molecules were generated based on the characterization results. Figure 10 illustrates the placement of an asphaltene molecule at the center of a 2.5 nm amorphous cell. Eight cells were synthesized to form a 5 nm × 5 nm × 5 nm supercell, which was filled with 350 n-heptane, 233 toluene, and 11 different ionic liquid molecules. The constructed supercell was then geometrically optimized and annealed. The annealing process was performed in the NVE ensemble, where the system was heated from 323.15 K to 823.15 K and subsequently cooled back to 323 K, with a relaxation pressure of 0.1 MPa. Following annealing, a 500 ps NPT ensemble was employed to achieve a reasonable density under the conditions of 323.15 K and 0.1 MPa. Subsequently, a 1000 ps NVT ensemble MD simulation at least was performed, at a temperature of 323.15 K.

## 5. Conclusions

Imidazolium chloride ionic liquids have been found to effectively reduce the viscosity of heavy crude oil. These ionic liquids interact with asphaltene molecules in crude oil, leading to their dispersion and a decrease in the size of asphaltene aggregates, ultimately resulting in a reduction in crude oil viscosity. Experiments and simulations confirm that the viscosity-reducing effect of ionic liquids on heavy crude oil is related to their ability to disperse asphaltenes. Experimental studies have demonstrated that 1-Dodecyl-3-methylimidazolium chloride ([C12-MIM]Cl) is efficient in reducing viscosity, exhibiting a 49.87% decrease when added at a concentration of 1500 mg/L. The asphaltene from Shengli crude oil was separated and characterized, and its solubility parameters were calculated based on the obtained molecular structures. It was found that imidazolium chloride ionic liquids and the isolated asphaltenes exhibited similar solubility parameters. The addition of 150 mg/L of [C12-MIM]Cl increased the initial deposition point of asphaltene from 63 vol% to 68 vol% and reduced the average size of asphaltene sediment particles from 79.35 μm to 48.54 μm. Using asphaltene models derived from characterization results, molecular dynamics simulations confirmed that ionic liquids effectively reduce the aggregation of asphaltene molecules, as evidenced by changes in the radial distribution function and aggregation patterns. Both experimental investigations and simulations have confirmed that alkyl chains of appropriate length in imidazolium chloride ionic liquids contribute to the dispersion of asphaltenes. Alkyl chains that are too short are weak in their ability to provide spatial resistance, and those that are too long result in poor solubility properties of the ionic liquid; thus, there is an optimum chain length for alkyl chains when designing ionic liquid viscosity reducers. The adsorption of imidazolium chloride ionic liquids onto the asphaltene surface through electrostatic and π–π interactions disrupts the spatial network formed by asphaltenes in the crude oil, thereby reducing the overall viscosity of the crude oil.

## Figures and Tables

**Table 1:** The results of average structure parameters of asphaltene.

Structural Parameters	Value
f_A_	0.57
f_N_	0.06
f_P_	0.37
σ	0.41
L	7.7
R_N_	7.11
R_A_	12.35
R_T_	19.46
usw	1089.3
Average molecular formula	C_71_H_78_S_3_NO_2_

f_A_ is the aromatic carbon fraction, f_N_ is the cycloalkane carbon fraction, f_P_ is the alkyl carbon fraction, σ is the substitution rates of carbon around aromatic ring systems, L is the average side chain carbon number, usw is the structural unit weight, R_N_ is the number of cycloalkane rings, R_A_ is the number of aromatic rings, and R_T_ is the total number of rings.

**Table 2:** Forms and percentages of O, S, and N in asphaltene.

Elements	Form	Binding Energy (eV)	Atomic Ratio (%)
O	C=O	531.33	8.72
	C-O	532.17	50.40
	COO-	532.58	40.88
S	Aliphatic-S	162.94	7.84
	Thiophenes	163.94	60.84
	Sulfoxide	165.12	31.32
N	Pyridinic-N	398.54	45.06
	Pyrrolic-N	400.11	54.94

**Table 3:** Calculation results of solubility parameters.

Material	δ_IL_ (MPa^1/2^)
Asphaltene	20.60
[BMIM]Cl	24.14
[OMIM]Cl	21.76
[C12-MIM]Cl	20.47
[C16-MIM]Cl	19.62

**Table 4:** Properties of heavy crude oil in Shengli.

Test	Results
Density (kg·m^−3^) at 20 °C	973.40
Viscosity (mPa·s) at 50 °C	15,000.00
Saturate and Aromatic (wt%)	53.42
Resins (wt%)	35.09
Asphaltene (wt%)	10.68

## Data Availability

Data are contained within the article or the Supplementary Materials.

## Supplementary Materials

The following supporting information can be downloaded at: https://www.mdpi.com/article/10.3390/molecules29051184/s1, Figure S1: Variation of absorbance of heavy crude oil solution with n-heptane volume fraction; Table S1. Relative molecular weight and elemental analysis results of asphaltenes; Figure S2. Asphaltene characterization results: (a) the ^1^H NMR spectrum, (b) the ^13^C NMR spectrum, (c) the infrared spectra; Table S2. Asphaltene ^1^H NMR results; Table S3. Asphaltene ^13^C NMR results; Figure S3. XPS characterization results; Table S4. Density of ionic liquids; Figure S4. Group decomposition of imidazole-based ionic liquids; Table S5. Molar attraction constants of functional groups ^[124]^; Figure S5. Effect of ionic liquids on particle size distribution of asphaltene sediment particles: (a) blank, (b) [BMIM]Cl, (c) [OMIM]Cl, (d) [C12-MIM]Cl, (e) [C16-MIM]Cl; Table S6. Contributions of atoms and functional groups in cations; Table S7. Contributions of atoms and functional groups in anions. References [42,43,44].
