# Supplementary material for: Experimental and Simulation Studies of Imidazolium Chloride Ionic Liquids with Different Alkyl Chain Lengths for Viscosity Reductions in Heavy Crude Oil: The Effect on Asphaltene Dispersion
Source: Molecules. 2024 Mar 6;29(5):1184. doi: 10.3390/molecules29051184 (PMC10935339; doi:10.3390/molecules29051184)
Supplement: Supplementary file 1 [file molecules-29-01184-s001.zip › molecules-2879292-supplementary.pdf]

Supporting information

Experimental and Simulation Studies of Imidazolium Chloride Ionic Liquids with Different Alkyl Chain Lengths for Viscosity Reductions in Heavy Crude Oil: The Effect on Asphaltene Dispersion

Chaoyue Xiang [1], Yangwen Zhu [2], Guanghao Liu [1], Tao Liu [3], Xinru Xu [1] and Jingyi Yang [1,*]

[1] International Joint Research Center of Green Energy Chemical Engineering, East China University of Science and Technology, Meilong Road 130, Shanghai 200237, China; y30210230@mail.ecust.edu.cn (C.X.); ghliuzz@163.com (G.L.); xrxu86@ecust.edu.cn (X.X.)

[2] Petroleum Exploration and Production Research Institute, SINOPEC, Beijing 100083, China; zhuyangwen.slyt@sinopec.com

[3] Shanghai Key Laboratory of Multiphase Materials Chemical Engineering, School of Chemical Engineering, East China University of Science and Technology, Shanghai 200237, China; liutao@ecust.edu.cn

* Correspondence: jyyang@ecust.edu.cn

Text S1

The initial deposition point of asphaltene method

The deposition of asphaltene was evaluated by measuring the absorbance using a UV spectrophotometer. The decrease in absorbance indicated the onset of asphaltene deposition. The initial point of asphaltene deposition was determined as the volume fraction of n-heptane at which the absorbance began to decrease. 9 mL of n-heptane:toluene mixtures (0:9, 1:8, 2:7, 3:6, 4:5, 5:4, 6:3, 7:2, 8:1, and 9:1 vol/vol), 0.6 mL of heavy crude oil toluene solution (30 g/L), and 0.4 mL of ionic liquid ethanol solution (25 g/L) were mixed and kept at 25°C for 24 hours. Subsequently, the absorbance of each solution was measured at 620 nm using a 752N UV-visible spectrophotometer. As can be seen in Fig. S1, the absorbance starts to decrease rapidly when the volume fraction of n-heptane exceeds 60% without the addition of ionic liquids. One curve is plotted for the points where the n-heptane volume fraction is less than 60% and another curve is plotted for the points where the n-heptane volume fraction is greater than 60%. From the intersection of the two curves, it can be concluded that the initial deposition point of asphaltenes in a heavy crude oil solution is 63%.

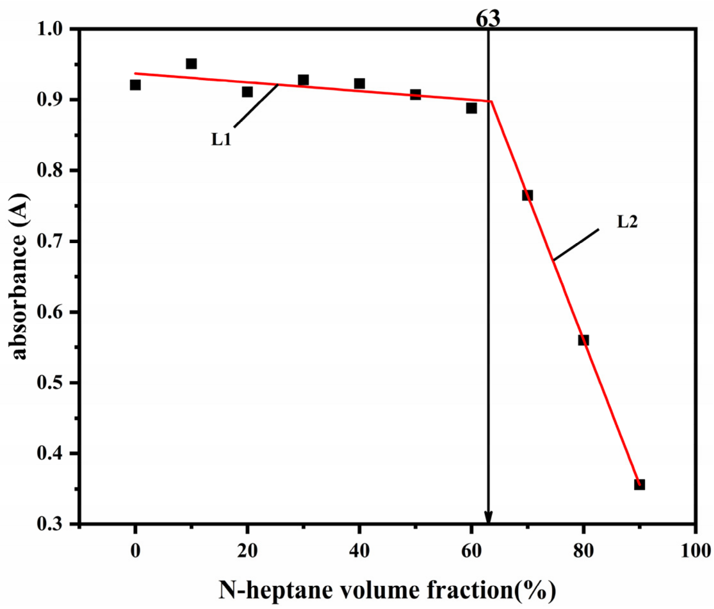

Figure S1. Variation of absorbance of heavy crude oil solution with n-heptane volume fraction

Text S2

Characterization of asphaltene structure

Table S1. Relative molecular weight and elemental analysis results of asphaltenes

	Result
**M_W**	5414
**C (%)**	79.46
**H (%)**	7.18
**S (%)**	3.14
**N (%)**	1.35
**O (%)**	8.87
**C/H**	0.92

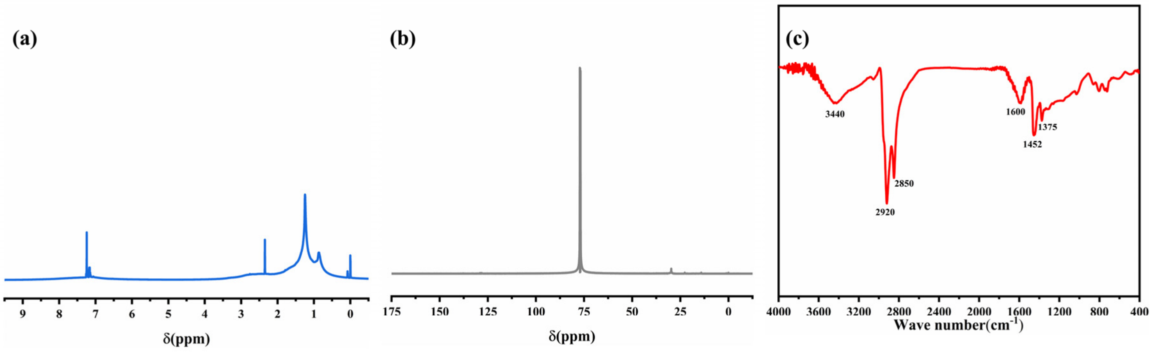

Figure S2. Asphaltene characterization results: (a) the ^{1}H NMR spectrum, (b) the ^{13}C NMR spectrum, (c) the infrared spectra

Table S2. Asphaltene ^{1}H NMR results

**Parameters**	**Types of hydrogen**	**peak ranges (ppm)**	**Ratios （%）**
**H_A**	Aromatic hydrogen	6.0~9.0	12.15
**H_α**	Alpha hydrogen in aromatic side chain	2.0~4.0	19.07
**H_β**	Beta hydrogen in aromatic side chain	1.0~2.0	51.40
**H_γ**	Gamma position and farther hydrogen in aromatic side chain	0.5~1.0	17.38

Table S3. Asphaltene ^{13}C NMR results

	Types of carbon	peak ranges (ppm)	Ratios (%)
**Aromatic carbon (100~150)**	Protonated aromatic carbon	100~127.1	18.03
	Aromatic bridgehead carbon	127.1~133	3.59
	CH_3 alkylated aromatic carbon	133~135	2.14
	cycloalkyl-arylene carbon	135~137	7.00
	≥C_2 alkylated aromatic carbon	137~150	8.89
**Saturated carbon (10~66)**	γ-CH_3	10~15	5.00
	β-CH_3	15~18	2.88
	α-CH_3，branched CH_3	18~22.5	6.29
	α-CH_2，β-CH_3，Aliphatic CH	22.5~37	25.55
	α-CH	37~66	20.63

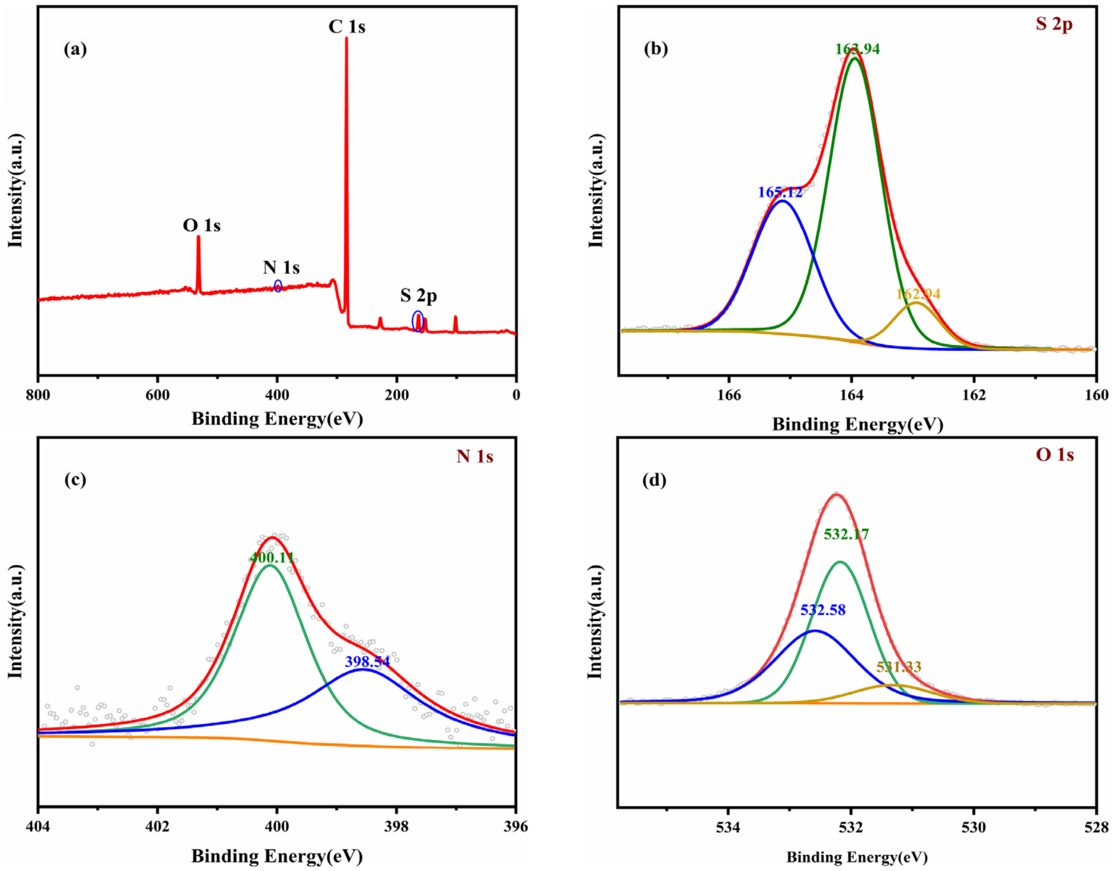

Figure S3. XPS characterization results

Text S3

Calculation method for solubility parameters

It is necessary to determine the density before calculating the solubility parameters of ionic liquids using the group contribution method. While experimental values for the densities of [BMIM]Cl and [OMIM]Cl are available, those for [C12-MIM]Cl and [C16-MIM]Cl are not[1,2]. Therefore, the latter must be calculated using the group contribution method [3]. To calculate the density of the ionic liquids, use Eq. S1, while Eq. S2 can be used to determine the molar volume. Finally, Eq. S3 is used to calculate dm, and the contribution values of the atoms and functional groups in the cations and anions of the ionic liquids can be found in Appendices 1 and 2, respectively.

$$\rho = \frac{M}{V_m} \quad \text{(S1)}$$

Where: ρ was the density of the ionic liquid, (g/m^3); M was the molecular mass of the ionic liquid,(g/mol); V_m was the molar volume, (m^3/mol).

$$V_m = dm + \frac{N_{at}}{100}(c + aT + bP) \quad (S2)$$

$$dm = V_0 + n_r V_r + \sum n_i V_{c_i} + \sum m_j V_{a_j} \quad (S3)$$

Where: V_o, V_r were constants; n_r denotes the total number of rings in the ionic liquid (including anions and cations); V_c, V_a were the contribution values of the functional groups in the cations and anions, respectively (check Appendices 1 and 2); n_i, m_j were the number of functional groups in the cations and anions, respectively; N_{at} was the total number of atoms in the ionic liquid; T was the temperature (K); P was the pressure (KPa).

The density value calculated using this method was denoted as ρ_{cal}, and the experimental value identified from the literature was denoted as ρ_{exp}, defining RE as the relative error between the calculated value ρ_{cal} and the experimental value ρ_{exp} as shown in Equation S4

$$RE = \frac{|\rho_{cal} - \rho_{exp}|}{\rho_{exp}} \quad (S4)$$

Where RE was the relative error; ρ_{cal} was the calculated value of density; ρ_{exp} was the experimental value of density.

Table S4 displays the calculated value ρ_{cal} of the ionic liquids, the experimental value ρ_{exp} obtained from the literature, and the corresponding relative error RE. It can be seen that the relative errors between the calculated ionic liquid density value ρ_{cal} and the experimental value ρ_{exp} are all within 1%. Therefore, we utilized the calculated values ρ_{cal} of the density for [C12-MIM]Cl and [C16-MIM]Cl and the experimental values ρ_{exp} of the density for the remaining ionic liquids in computing the ionic liquid solubility parameters.

Table S4. Density of ionic liquids

Ionic liquid	ρ_{cal} (g/cm^3)	ρ_{exp} (g/cm^3)	RE(%)
**[BMIM]Cl**	1.0881	1.0816	0.60
**[OMIM]Cl**	1.0118	1.0096	0.22
**[C12-MIM]Cl**	0.9704	/	/
**[C16-MIM]Cl**	0.9444	/	/

We employed the group contribution method developed by Trevor K. Carlisle et al. to determine the solubility parameters of the ionic liquids[4]. This involved

computing the molar volume of the ionic liquid using Equation S5 and, subsequently, utilizing Equation S6 to derive its solubility parameter.

$$V_m = \frac{M}{\rho} \qquad \text{(S5)}$$

Where V_m was the molar volume of the ionic liquid (cm^3/mol); M was the molar mass of the ionic liquid (g/mol); ρ was the density of the ionic liquid (g/cm^3).

$$\delta_{IL} = \frac{\sum_j F_j}{V_m} \qquad \text{(S6)}$$

Where δ_{IL} was the solubility parameter of the ionic liquid ($MPa^{1/2}$); F_j was the molar attraction constant of the ionic liquid group ($MPa^{1/2} \cdot cm^3 \cdot mol^{-1}$); V_m was the molar volume of the ionic liquid (cm^3/mol).

To categorize the imidazole ionic liquids used in this study, they were first divided based on their respective functional groups and structures, illustrated in Fig. S4. The imidazole ionic liquids were then separated into three parts, with R_1 and R_2 comprising one part each and the imidazole ring and anion forming the third part. Analysis of the imidazolium ionic liquids in this study revealed that the main functional groups of R_1 and R_2 were $-CH_3$, $-CH_2-$, and phenyl, each displaying varying molar attraction constants as outlined in Table S5. The molar attraction constants of the imidazole ring and Cl- were determined through back-calculation using the solubility parameter of 1-butyl-3-methylimidazolium chloride salt [BMIM]Cl (δ_{IL}=24.14$MPa^{1/2}$).

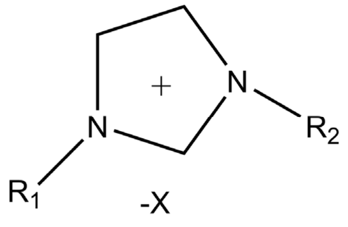

Figure S4. Group decomposition of imidazole-based ionic liquids

Table S5. Molar attraction constants of functional groups [124]

Group	F（$MPa^{1/2} \cdot cm^3 \cdot mol^{-1}$）
**$-CH_3$**	303
**$-CH_2-$**	269
**Phenyl**	1400

Text S4

Particle Size of asphaltene

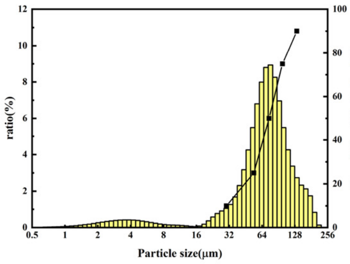

(a)Blank

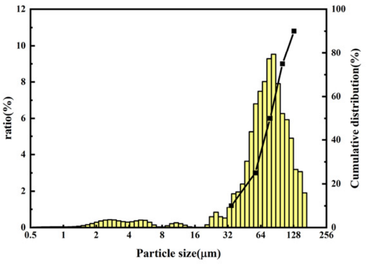

(b) [BMIM]Cl

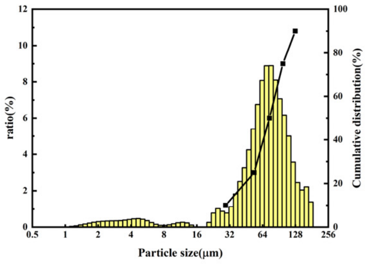

(c) [OMIM]Cl

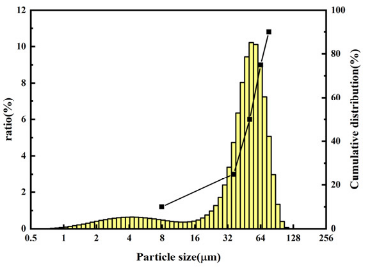

(d) [C12-MIM]Cl

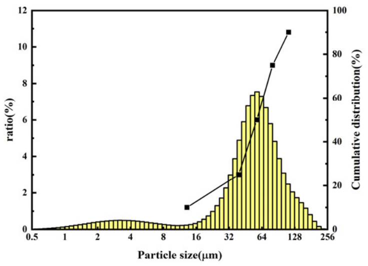

(e) [C16-MIM]Cl

Figure S5. Effect of ionic liquids on particle size distribution of asphaltene sediment particles: (a) blank, (b) [BMIM]Cl, (c) [OMIM]Cl, (d) [C12-MIM]Cl, (e) [C16-MIM]Cl

Table S6. Contributions of atoms and functional groups in cations[3]

Group		$V_c(cm^3/mol)$
**Atomic contribution**		
**N**		12.0965
**C**		12.2105
**H**		0.9746
**O**		0.6759
**F**		0.7919
**Si**		27.2494
**P**		14.5812
**S**		17.5598
**Br**		5.9869
**Fe**	m=1[a]	−81.9660
**Cl**	m=1	82.7637
**I**		20.4978
**Functional group contribution[b]**		
**=C< (ring)[c]**		−3.4898
**NH (ring)**		9.3246

**>CH–[d]**		2.7969
**CO**		14.3775
**COO**		5.4468
**OH**		2.0058
**C≡N**		−11.8741
**COOH**		11.9135
**Rn[e]**	n>2	0.0851

a：m was the number of times the atom appears in the cation;

b：Those groups not represented by the symbols > or - (e.g., NH (ring), CO, COO) can also be connected to atoms (including H) or functional groups by double or single bonds;

c：（ring）；It indicates that the group is in the ring structure;

d：The > or - symbols indicate groups linked to functional groups or atoms other than H;

e：n-alkyl chains with n carbon atoms, e.g. butyl or pentyl.

Table S7. Contributions of atoms and functional groups in anions[3]

Group	$V_a(cm^3/mol)$
**Atomic contribution**	
**Cl**	21.6536
**Br**	29.6404
**I**	46.3084
**O**	6.2398
**B**	20.3946
**F**	6.2740
**N**	8.4436
**S**	23.3129
**C**	11.1926
**P**	25.6053
**H**	1.2163
**Al**	18.7904
**Nb**	42.3397
**Ta**	39.0276
**W**	32.3795
**Ga**	19.3993
**Fe**	18.6362
**In**	26.2812
**Functional group contribution**	
**CN**	1.7655

**=C<(ring)[a]**	-1.1086
**CO**	-12.6131
**COO**	8.0128
**COOH**	−22.7409
**OH**	−6.6065

a：(ring)；It indicates that the group is in the ring structure.

References

1. He, R.-H.; Long, B.-W.; Lu, Y.-Z.; Meng, H.; Li, C.-X., Solubility of Hydrogen Chloride in Three 1-Alkyl-3-methylimidazolium Chloride Ionic Liquids in the Pressure Range (0 to 100) kPa and Temperature Range (298.15 to 363.15) K. *Journal of Chemical & Engineering Data* **2012,** 57, (11), 2936-2941.
2. Mac Dowell, N.; Llovell, F.; Sun, N.; Hallett, J. P.; George, A.; Hunt, P. A.; Welton, T.; Simmons, B. A.; Vega, L. F., New experimental density data and soft-SAFT models of alkylimidazolium ([C(n)C(1)im](+)) chloride (Cl(-)), methylsulfate ([MeSO(4)](-)), and dimethylphosphate ([Me(2)PO(4)](-)) based ionic liquids. *J Phys Chem B* **2014,** 118, (23), 6206-21.
3. Taherifard, H.; Raeissi, S., Estimation of the Densities of Ionic Liquids Using a Group Contribution Method. *Journal of Chemical & Engineering Data* **2016,** 61, (12), 4031-4038.
4. Carlisle, T. K.; Bara, J. E.; Gabriel, C. J.; Noble, R. D.; Gin, D. L., Interpretation of CO2 Solubility and Selectivity in Nitrile-Functionalized Room-Temperature Ionic Liquids Using a Group Contribution Approach. *Industrial & Engineering Chemistry Research* **2008,** 47, (18), 7005-7012.
